# Difference in the Electromyographic Onset of the Deep and Superficial Multifidus during Shoulder Movement while Standing

**DOI:** 10.1371/journal.pone.0122303

**Published:** 2015-04-07

**Authors:** Teppei Abiko, Ryota Shimamura, Daisuke Ogawa, Yoko Abiko, Masaki Hirosawa, Natsumi Momose, Wataru Tsuchihashi, Takaharu Suzuki, Hitoshi Takei

**Affiliations:** 1 Department of Physical Therapy, Faculty of Health Sciences, Kyoto Tachibana University, Yamashina-ku, Kyoto, Japan; 2 Division of Physical Therapy Science, Faculty of Human Sciences Graduate School of Human Health Sciences, Tokyo Metropolitan University, Arakawa-ku, Tokyo, Japan; 3 Division of Physical Therapy, Tokyo Metropolitan Rehabilitation Hospital, Sumida-ku, Tokyo, Japan; 4 Department of Physical Therapy, Faculty of Human Health Sciences, Mejiro University, Saitama-shi, Saitama, Japan; 5 Division of Physical Therapy, Senri Chuo Hospital, Toyonaka-shi, Osaka, Japan; 6 Center for Biosciences and Informatics, Graduate School of Fundamental Science and Technology, Keio University, Yokohama-shi, Kanagawa, Japan; Institut Pluridisciplinaire Hubert Curien, FRANCE

## Abstract

Based on the current literature, it remains unclear whether electromyographic onset of the deep fibers of the multifidus (DM) is dependent on the direction of shoulder movement and the position of the center of foot pressure (CFP). In the present study, we re-examined the electromyographic onset of the DM during shoulder flexion and extension and investigated the influence of the CFP position before arm movement. Intramuscular and surface electrodes recorded the electromyographic onset of the DM, superficial fibers of the multifidus (SM), rectus abdominis, and anterior and posterior deltoid. Eleven healthy participants performed rapid, unilateral shoulder flexion and extension in response to audio stimuli at three CFP positions: quiet standing, extreme forward leaning, and extreme backward leaning. It was found that the electromyographic onset of the DM and SM relative to the deltoid was dependent on the direction of arm movement. Additionally, of all electromyographic onsets recorded, only that of the DM occurred earlier in the extreme forward leaning position than in the extreme backward leaning position during shoulder flexion. These results suggest that the electromyographic onset of DM was influenced by the biomechanical disturbance such as shoulder movement and CFP position.

## Introduction

Anticipatory postural adjustment (APA) is an involuntary and automatic adjustment generated during disturbance in a predictable posture [1–4]. APA occur within the range of -100 ms to +50 ms from the start of activity in a focal muscle [4,5]. These adjustments in a postural muscle help maintain the center of gravity and base of support [6] as well as stabilize the vertebral segments [7]. During unilateral shoulder movement, the central nervous system responds to the postural disturbance by activating muscles that allow movement to the opposite side in an attempt to maintain posture [4,8–10]. Therefore, when the shoulder is flexed quickly, the erector spinae is activated prior to the initiation of muscular activity in the anterior deltoid. On the other hand, when the shoulder is extended, the abdominal muscles are activated prior to the posterior deltoid. Therefore, APA is dependent on the direction of the disturbance such as those caused by shoulder movement.

According to some studies, activation of the transversus abdominis (TrA) often generates feedforward activity that occurs prior to the focal muscle activity regardless of the direction of the shoulder movement [5,11]. Similarly, it has also been reported that the deep fibers of the lumbar multifidus (DM) are not dependent on the direction of arm movement, unlike the superficial fibers of the lumbar multifidus (SM) [11]. Furthermore, biomechanical data has revealed that there is a difference in the functions of superficial and deep muscles [12]. Superficial muscles control the movement of the lumbar vertebrae, allowing for large arm movements, whereas deep muscles are small and control the intervertebral movements [12]. These results suggest that local muscles such as the TrA and DM are intersegmental motion stabilizers. Therefore, in this paper, the term “APA” can be interpreted as a direction-dependent activity, and the term “stabilizer” can be interpreted as a direction-independent activity.

However, some studies have suggested that the electromyographic (EMG) onset of the deep fibers of back muscles is dependent on the direction of shoulder movement [13,14]. The study by MacDonald et al. [13] has already shown that the EMG onset of the DM is independent of the direction of shoulder movement. The study by Lee et al. [14] demonstrated that the EMG onset of thorax multifidus does not depend on the direction of shoulder joint movement. These studies by MacDonald et al. and Lee et al. have different conclusions and methodologies; therefore, re-examination is warranted to verify whether the deep fibers of the multifidus are dependent on the direction of shoulder movement.

Muscular activity before movement, center of gravity, posture, mental state, movement task, fatigue, pain, and proprioceptive information are some of the factors that influence APA [4,9,10,14–25]. Previously, studies have been performed on deep abdominal muscles in relation to different movement tasks and presence of pain. Hodges et al. [9,19,20] indicated that the TrA does not act in a feedforward manner in patients with low back pain. Furthermore, they mentioned that selective exercises of the TrA revived the feedforward activity and reduced low back pain in patients [26,27]. MacDonald et al. [13] evaluated the EMG onset of the DM in subjects with a history of low back pain. In their results, the EMG onset of the DM was significantly delayed on the painful side compared with that on the non-painful side, which suggested DM dysfunction. However, in that study, the posture and center of foot pressure (CFP) position, which are considered to influence APA, were not examined in detail [18]. In general, many patients with low back pain have malalignment of the lumber spine and pelvis [28,29]; therefore, EMG onset of the DM may be delayed because of malalignment.

Cord and Nashner [30] suggested that the equilibrium state and standing posture influence APA at the beginning of focal movement in the upper limbs. Furthermore, Fujiwara et al. [18] and Benvenuti et al. [23] clarified that when the CFP position is set in a more forward leaning position than in the quiet standing (QS) posture, muscle activity is initiated earlier in the erector spinae. These studies used a surface EMG approach and did not measure the DM using intramuscular electromyography. Based on the possibility of the DM being dependent on the direction of the biomechanical disturbance, we hypothesized that the EMG onset of the DM is influenced by the CFP position, as in the case of erector spinae.

The present study aimed to investigate whether the EMG onsets of DM and SM change according to the direction of shoulder movement and the CFP position. We hypothesized that the EMG onset of the DM would be dependent on the direction of shoulder movement. Additionally, based on the notion that the EMG onset of DM is dependent on biomechanical perturbation, we hypothesized that the EMG onset of the DM would occur earlier in the extreme forward leaning (EFL) position than in the extreme backward leaning (EBL) position. This study will provide useful information in the treatment of patients with a delayed EMG onset of the DM. It may help in improving the EMG onset of the DM by providing instructions regarding the appropriate direction of shoulder movement and the CFP position.

## Materials and Methods

### Subjects

Eleven healthy men (mean ± SD; age, 30.7 ± 5.5 [range, 24–38] years; height, 172.7 ± 4.3 cm; body mass, 62.2 ± 5.1 kg) with no neurological or muscular disorders participated in the experiment. The exclusion criterion was as follows: poor pelvic alignment while standing, with an angle >5° between the vertical line and the line connecting the anterior superior iliac spine and pubic symphysis.

Following an explanation of the experimental protocol, all participants provided written informed consent, and the study was performed in accordance with the Declaration of Helsinki. This study was approved by the Ethics Committee of Tokyo Metropolitan Rehabilitation Hospital and Tokyo Metropolitan University.

### Apparatus

#### EMG acquisition

EMG signals of selected trunk and thigh musculature were recorded using a combination of intramuscular fine-wire and surface electrodes with the TRAIS System (DKH Inc., Tokyo, Japan). The EMG signals from the left SM and DM were recorded using fine-wire bipolar electrodes fabricated from two strands of urethane-coated stainless-steel wire (diameter, 0.05 mm; Unique Medical Co, Ltd, Tokyo, Japan). The fine-wire was threaded into hypodermic needles (23 gauge × 60 mm), with 1 mm of urethane removed and the tips bent back to form 1- to 2-mm hooks. Wire electrodes were sterilized by STERRAD (Sterrad^@^ NX; Johnson and Johnson Corp, Tokyo, Japan). The intramuscular electrodes were inserted with the participants in the sitting position. The L4 vertebral lamina and the target muscles were clearly identified. The electrode that recorded the DM EMG was inserted 40 mm lateral to the midline and directed anteromedially until the tip of the needle reached the most medial aspect of the L4 lamina [11]. The electrode that recorded the SM EMG was inserted 20 mm lateral to the midline and directed anteromedially until the tip of the needle was visualized in the superficial back muscle [11]. After needle removal, gentle traction of the wires under ultrasound visualization confirmed the position of each electrode. Participants reported only mild transient discomfort during the insertions, and if any significant discomfort was reported after the needle removal, the electrode was removed, and a new electrode was inserted. This was performed to ensure minimum pain during the experiment.

In addition, parallel to the muscle fibers, with a center-to-center distance of 2 cm, pairs of disposable Ag/AgCl surface electrodes (Vitrode F-150S; Nihon Kohden Corporation, Tokyo, Japan) were attached to the following muscles: left rectus abdominis (RA, 3 cm lateral to the umbilicus) and the right anterior and posterior deltoid. A reference electrode was placed over the left ulnar styloid process. Before the surface electrodes were attached, the skin was shaved and rubbed with a skin abrasive and alcohol to reduce the skin impedance to a level below 5 kΩ, and all electrode pairs were tested with volitional activation.

EMG signals were collected at 1 kHz and bandpass filtered between 15 and 450 Hz. All data were exported for analysis with Matlab 6.5 (Mathworks, Natic, MA, USA).

#### Platform acquisition

All measurements were performed with the participants standing on a force platform (Kistler Instruments Inc., Amherst, NY, USA). The platform was used to record the CFP positions in the anterior-posterior direction. Signals were sampled at 1000 Hz.

### Experimental protocol

#### Positions

All measurements were taken with the participants standing barefoot, with their feet 15 cm apart and parallel on the force platform. This foot position was marked on the platform and reproduced across trials. The mean CFP was initially measured when the participants maintained a QS posture with their arms by their sides. Next, the mean CFP position was measured during an EFL posture in which the subjects moved the CFP to the maximum forward position maintaining their posture. The participant gradually leaned forward from the QS posture for more than 3 s, pivoting at the ankles, while the alignment of the rest of the body was maintained; this EFL posture was then maintained. The most anterior CFP position was adopted as the EFL position. A similar procedure was used to measure the CFP position in an EBL position, for which the participants leaned backward by pivoting at the ankles. The participants maintained the QS, EFL, and EBL standing positions and the respective CFP positions before arm movement.

The QS, EFL, and EBL positions were randomly set as the initial position. To reproduce the CFP position accurately, continuous visual feedback was provided on the center of pressure trajectory by the monitor placed 1.0 m in front of the subject at eye level. The subjects practiced performing the arm task several times before initiation of the actual experiment.

#### Task

The participants stood relaxed with their arms by their sides. They were instructed to rapidly flex the right shoulder up to 90° or extend the shoulder up to 45° with their elbows straight, in response to an auditory cue. They were instructed to react as quickly as possible to the cue by rapidly moving their arm in the correct direction. The auditory cue occurred randomly, between 3- and 7-s intervals. Several practice trials were performed prior to data collection to ensure that the reaction time was as consistent as possible, given the variable reaction time in humans, and that the correct movement of the arm was performed in response to each auditory cue. Five repetitions were performed in each direction.

### Data analysis

The EMG onset for each muscle was visually identified as the point at which the activity increased above the baseline. EMG onset detection was aided by the ability to visualize single motor units, and therefore, detected the recruitment of new motor units as an indicator of the onset of EMG activity. Recordings were displayed in a random order, without reference to other muscles or events. The investigator was blind to the identity of the muscle being evaluated. There was no difference in the different intramuscular recordings that could alert the investigator to the identity of the muscle. This method of EMG analysis is reliable and valid.

The onsets of the DM, SM, and RA relative to that of the deltoid were used for analysis. Trials were excluded if the onset of back muscle activity occurred either 100 ms before or 200 ms after that of the deltoid, because activations outside of those times are unlikely to be related to the arm movement [4]. Less than 6.8% of the trials were excluded based on these criteria.

### EMG activity

The mean onset of the EMG burst of postural muscles relative to the deltoid was obtained for each standing position (QS, EFL, and EBL) and adopted as the representative value for the participant and used in subsequent analyses [5,11,12].

The CFP position in the anterior-posterior direction from the heel relative to the total foot length was defined as the percentage of foot length (%FL) [18]. The %FL was measured between -300 ms to -250 ms relative to the EMG onset of the deltoid, so that it did not affect the APA.

### Statistical analysis

The EMG onsets of each trunk muscle relative to that of the deltoid were averaged for all trials. To confirm that the CFP at the three initial positions were different, one-way analysis of variance with repeated measures was used to compare the CFP. Post-hoc analysis was performed using the Bonferroni test.

We used the two-way repeated measures analysis of variance with the main effect being direction (flexion and extension) and CFP positions (QS, EFL, and EBL) for each muscle. Post-hoc analysis was performed using the Bonferroni test.

Alpha level was set at 0.05. All statistics were calculated using SPSS version 19 (IBM, Tokyo, Japan).

## Results

### Effect of movement direction

**Fig 1:**
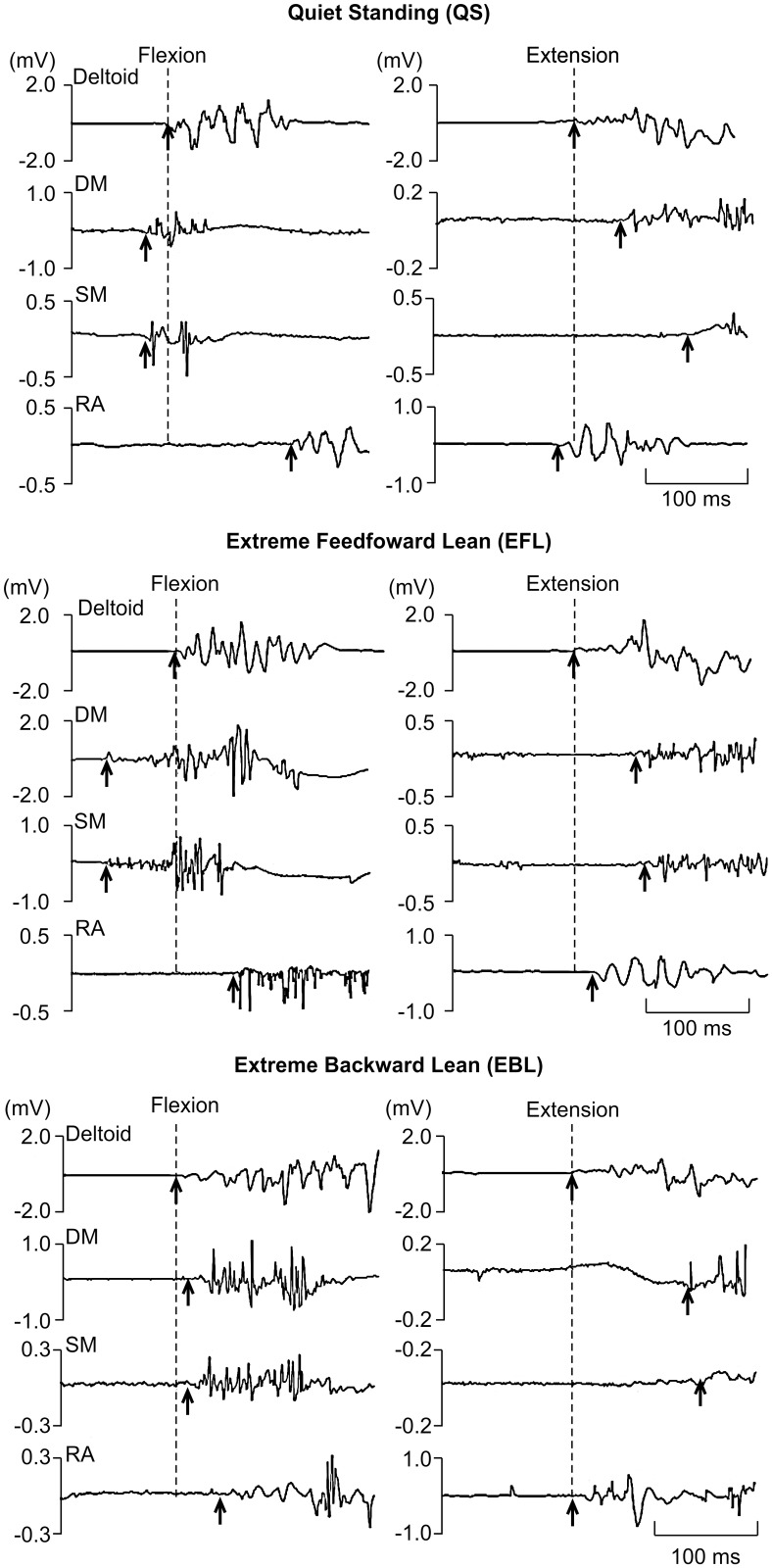
Raw electromyographic data for shoulder movements in three standing positions (QS, EFL, and EBL). The left panels show data for shoulder flexion, and right panels show data for shoulder extension. Panels show electromyographic data for anterior or posterior deltoid as focal muscle, deep (DM) and superficial (SM) fibers of lumbar multifidus and rectus abdominis (RA). The dashed vertical lines represent the EMG onset of deltoid, and the arrows represent the EMG onset of each muscle.

The EMG onset of the DM occurred earlier with shoulder flexion than with shoulder extension at each CFP position (Interaction: direction × CFP, *P* < 0.01; post hoc, *P* < 0.01) (Fig 1, Tables 1 and 2). The EMG onset of the SM occurred earlier during shoulder flexion than during shoulder extension at all CFP positions (main effect: direction, *P* < 0.01) (Table 1). In contrast, there was no difference in the EMG onset of the RA (Table 1).

**Table 1:** Two-way repeated measures analysis of variance for comparisons between the direction of shoulder arm movement and the center of foot pressure (CFP) position while standing in each muscle.

	DM		SM		RA	
	*F* values	*P* values	*F* values	*P* values	*F* values	*P* values
Direction	132.20	0.00	36.20	0.00	3.32	0.10
CFP	1.42	0.28	0.27	0.74	0.22	0.78
Direction × CFP	7.13	0.00	0.34	0.72	0.18	0.84

DM, Deep fibers of lumbar multifidus; SM, Superficial fibers of lumbar multifidus; RA, Rectus abdominis

**Table 2:** Comparison of the EMG onset of each muscle relative to the deltoid (mean ± SEM, ms) according to the direction and center of foot pressure (CFP) position.

CFP Position		QS	EFL	EBL
DM	Flex	-2.9 ± 7.2^†^	-32.0 ± 8.5* ^†^	5.7 ± 6.0^†^
	Ext	82.4 ± 7.8	96.4 ± 10.0	93.1 ± 16.0
SM	Flex	-4.0 ± 5.9	14.5 ± 16.6	-1.4 ± 8.3
	Ext	59.2 ± 12.4	61.6 ± 15.4	58.7 ± 11.1
RA	Flex	71.0 ± 20.1	67.3 ± 19.6	68.2 ±14.3
	Ext	30.6 ± 9.3	37.2 ± 10.1	26.4 ± 4.2

*The EMG onset of the DM in the EFL position occurred earlier than that in the EBL position with shoulder flexion (post hoc: P = 0.01).

^†^The EMG onset of DM occurred earlier with shoulder flexion than with shoulder extension at each CFP position (post hoc: *P* < 0.01).

DM, Deep fibers of the lumbar multifidus; SM, Superficial fibers of lumbar multifidus; RA, Rectus abdominis; Flex, shoulder flexion; Ext, shoulder extension; QS, Quiet standing; EFL, Extreme forward leaning; EBL, Extreme backward leaning

### Effect of the CFP position

**Fig 2:**
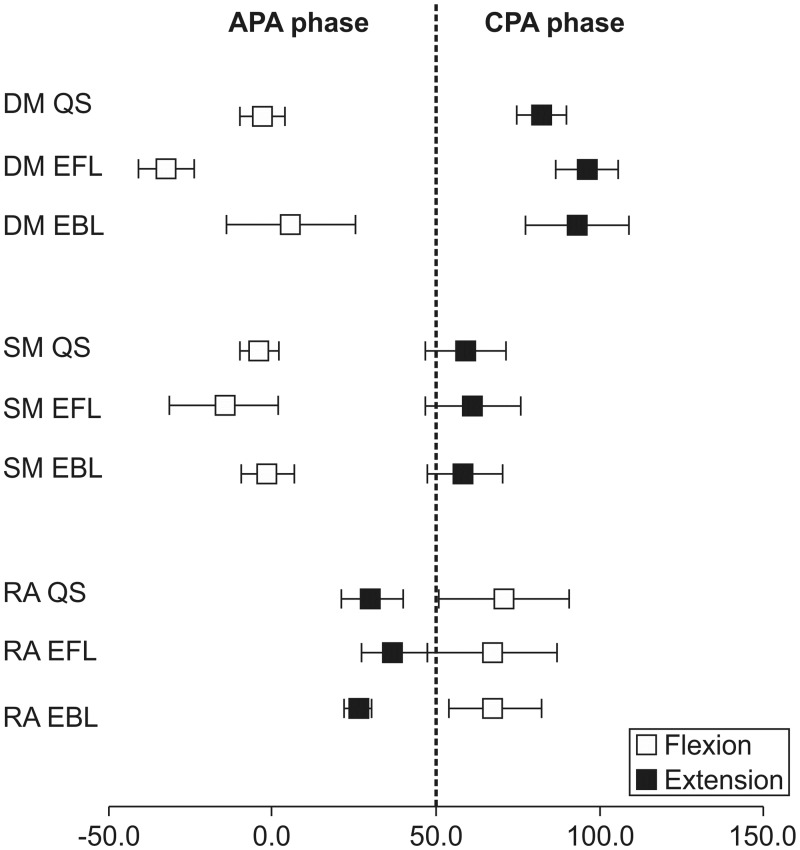
Mean+SEM (ms) EMG onset of each trunk muscle relative to that of the deltoid. Data during flexion (open squares) and extension (close squares) are shown. The dashed vertical line represents the APA phase or CPA phase. APA, Anticipatory postural adjustments; CPA, Compensatory postural adjustments; DM, Deep fibers of the lumbar multifidus; SM, Superficial fibers of the lumbar multifidus; RA, Rectus abdominis; Flex, Arm flexion; Ext, Arm extension; QS, Quiet standing; EFL, Extreme forward leaning; EBL, Extreme backward leaning; EMG, Electromyography.

The %FL of QS, EFL, and EBL were significantly different (Table 3). When the different CFP positions were compared, the EMG onset of the DM occurred earlier in the EFL position than in the EBL position only with shoulder flexion (Interaction: direction × CFP, *P* < 0.01; post hoc, *P* = 0.01) (Tables 1 and 2). The onsets of the SM and RA were not different between the CFP positions with shoulder flexion and extension. This indicates that only the EMG onset of the DM was influenced by the CFP position (Fig 2).

**Table 3:** The center of foot pressure position in the anteroposterior direction (% foot length [%FL]) during quiet standing (QS), extreme forward leaning (EFL), and extreme backward leaning (EBL).

	QS	EFL	EBL
%FL	43.6% ± 1.3%*	82.2% ± 1.9%*	27.1% ± 1.3%*

Data are shown as mean ± SEM.

*The %FL of QS, EFL, and EBL were significantly different (all comparisons, *P* < 0.01)

## Discussion

The results of this study indicate that the activation of the DM and SM depends on the direction of movement of the shoulder when the maintaining posture is challenged by voluntary arm movement. In addition, the EMG onset of only the DM occurred earlier in the EFL position than in the EBL position. These findings demonstrate that the EMG onset of the DM is influenced by biomechanical disturbance.

Both the DM and SM showed feedforward activity relative to the deltoid during shoulder flexion. However, during shoulder extension, they acted outside of the feedforward activity, or in other words, they did not show feedforward activity (Fig 1). Generally, during rapid shoulder flexion, the CFP is expected to move forward, which is counteracted by activation of the back muscles that move it backward [4]. On the other hand, during arm extension, the CFP moves backward secondary to the disturbance from the arm movement. Here, the back muscles do not need to be active to maintain the CFP in the base of support, and therefore do not show feedforward activity. Furthermore, many studies on the influence of biomechanical load on the trunk have reported that flexion moments occur in the trunk during shoulder flexion; therefore, trunk extensor activity acts as an APA against these moments [4,18,23,24,31,32]. Trunk muscle activity for postural adjustments during shoulder flexion is an APA that acts in the range of -100 ms to +50 ms from the start of focal muscle activity, and the following abdominal muscle and back muscle activity in the range of +50 ms to +200 ms for compensatory postural adjustments (CPA) is not voluntary but needs sensory feedback signals [15,33,34]. The role of CPA is also to restore the position of the center of the mass after a perturbation has already occurred [35,36]. Our research shows that the activity of the DM and SM serves as an APA during shoulder flexion, and as a CPA during upper limb extension. Furthermore, the EMG onsets of DM and SM are significantly different between shoulder flexion and extension. These results may indicate that the DM functions in a direction-dependent manner to maintain the CFP and standing posture.

Previous research presumed that long longitudinal muscles such as the erector spinae control the CFP and maintain spinal posture, and since short muscles such as the multifidus have disadvantages in torque, their role in CFP control and maintenance of spinal posture is limited, and they are mainly responsible for controlling intervertebral motions [12]. Based on this characteristic of the DM, our results may indicate that the DM control the CFP and standing posture indirectly to control the micro-motion between the vertebral bodies. Superficial back muscles therefore control the CFP and standing posture directly and efficiently. Thus, it is possible that this DM function of controlling vertebral micro-motion is a direction-dependent activity.

The direction-dependent activity of the DM is attributed to their muscular arrangement. The transversus abdominis and diaphragm as local trunk muscles, which do not depend on the direction of the shoulder movement, by virtue of their arrangement, surround the axes of movement in flexion and extension, lateral flexion, and rotation [37]. On the other hand, the multifidus is arranged such that it functions only during extension in the sagittal plane [12]. Therefore, the DM show feedforward activity during shoulder flexion and act outside of the feedforward activity during shoulder extension.

The EMG onset of the DM occurred significantly earlier in the EFL position than in the EBL position. It is reported that with shoulder flexion, the CFP is predicted to move forward; therefore, the activity of back muscles starts prior to activity of the deltoid in order to move the CFP back in the base of support [4]. In the EFL position, the CFP may no longer move forward; therefore, the EMG onset of the DM may need to occur earlier to move the CFP back. Additionally, compared with shoulder flexion in the EBL position, when the shoulder is flexed in the EFL position, a greater degree of back muscle activity may be required to maintain posture and the CFP in the base of support. The lumbar spine may experience more stress while maintaining the standing position during shoulder flexion in the EFL than in the EBL position, and the CFP position may need to be shifted back. Therefore, the DM may stabilize the lumbar spine quickly to enable production of the torque by the superficial back muscle.

Furthermore, it is possible that the EMG onset of the DM affects not only the movement of the CFP and maintenance of the standing posture, but also the activity of the DM before shoulder movement. Fujiwara et al. [18] found a significant correlation between muscular activity and EMG onset latency of the erector spinae during rapid bilateral upper limb flexion. It is suggested that as a result of change in the afferent stimuli from muscles, the trunk and lower limb muscles are activated, and these play a significant role in maintaining the standing position. Our study may presume that the increase in the activity of the back muscles to maintain the EFL position was greater than that required to maintain the EBL position, which implies that the proprioceptor input differs depending on the strength of muscle activity. As a result, it is possible that the EMG onset of the DM is earlier in the EFL than in the EBL position.

Unlike the EMG onset of the DM, the EMG onset of the SM did not significantly differ at any CFP position. In other words, the DM is more easily influenced by the CFP than the SM, which clarified the functional difference between DM and SM. The DM are rich in proprioceptors sensitive to pressure and deviation of the intervertebral joint [38,39]. In our study, it is considered that changing the posture to EFL increased the muscular activity and pressure on the intervertebral joint, and this increased the proprioceptive sensitivity and changed the EMG onset of the DM. Therefore, our results suggest the possibility that the DM have the function of finely adjusting the onset of muscle activity even within the range of feedforward activity because of the significant difference between the EMG onset of the DM and SM.

This study had limitations that need to be acknowledged. Here, we were not able to measure kinematic data because our laboratory was not equipped with a digital goniometer and motion analysis system. Additionally, we did not examine bilateral trunk muscle responses and the amplitude of muscle activity. If the timing and amplitude of bilateral multifidus muscles are similar, the lumbar spine may be fixed without micro-motion, whereas increase in unilateral multifidus activity will cause side flexion or rotation of the lumbar spinal segment. Therefore, by bilateral muscle activation, we can determine the spinal stability and influence of the vertical and coronal planes and not just the sagittal plane. In addition, the DM activation patterns in patients with low back pain during change in the CFP position still need to be analyzed in future studies.

The results of the present study have clinical implications for the management of patients with low back pain who do not show rapid contraction of the DM. To restore the onset of the DM, these patients might be instructed to flex their shoulder quickly in the EFL position, with the pelvis in the neutral position, which will enable an earlier activation of the deep fibers. Further investigation is required for a thorough study of this concept in patients with low back pain.

## Conclusion

The present study revealed that the induced shoulder movement and CFP position affect the APA. There are significant differences in the EMG onset latency of the DM and SM depending on the direction of shoulder movement. Additionally, the EMG onset of only the DM occurred earlier in the EFL position than in the EBL position. This result shows that there is a functional difference between the DM and SM. It may also suggest that the DM is highly sensitive and that the onset of DM activity enables change in the initial position prior to initiation of arm movement.
