# Correction: Difference in the Electromyographic Onset of the Deep and Superficial Multifidus during Shoulder Movement while Standing

**DOI:** 10.1371/journal.pone.0133333

**Published:** 2015-07-15

**Authors:** Teppei Abiko, Ryota Shimamura, Daisuke Ogawa, Yoko Abiko, Masaki Hirosawa, Natsumi Momose, Wataru Tsuchihashi, Takaharu Suzuki, Hitoshi Takei

There are multiple errors in the second and third sentences of the third paragraph of the Introduction. The correct sentences are: The study by MacDonald et al. [13] has already shown that the EMG onset of the DM is dependent on the direction of shoulder movement. The study by Lee et al. [14] demonstrated that the EMG onset of thorax multifidus does depend on the direction of shoulder joint movement.
